# The Impact of Nature Experience on Willingness to Support Conservation

**DOI:** 10.1371/journal.pone.0007367

**Published:** 2009-10-07

**Authors:** Patricia A. Zaradic, Oliver R. W. Pergams, Peter Kareiva

**Affiliations:** 1 Red Rock Institute, Inc., Bryn Mawr, Pennsylvania, United States of America; 2 Department of Biological Sciences, University of Illinois at Chicago, Chicago, Illinois, United States of America; 3 The Nature Conservancy, Seattle, Washington, United States of America; University of Pretoria, South Africa

## Abstract

We hypothesized that willingness to financially support conservation depends on one's experience with nature. In order to test this hypothesis, we used a novel time-lagged correlation analysis to look at times series data concerning nature participation, and evaluate its relationship with future conservation support (measured as contributions to conservation NGOs). Our results suggest that the type and timing of nature experience may determine future conservation investment. Time spent hiking or backpacking is correlated with increased conservation contributions 11–12 years later. On the other hand, contributions are negatively correlated with past time spent on activities such as public lands visitation or fishing. Our results suggest that each hiker or backpacker translates to $200–$300 annually in future NGO contributions. We project that the recent decline in popularity of hiking and backpacking will negatively impact conservation NGO contributions from approximately 2010–2011 through at least 2018.

## Introduction

A review of recent trends suggests that current public support for conservation has a narrow base, and that existing support is not strong enough to make conservation or the environment a high priority [1], [2]. A 2004 survey of the electorate determined that Americans are increasingly indifferent to environmental issues, with the environment ranking lower than most other voter priorities [1], [2], [3]. At the same time there has been a well documented trend towards reduced nature-based recreation as measured on a per capita basis [4]. Finally, populations globally and within the US are increasingly urban, meaning there simply is less contact with nature in any form [5]. All of these trends combine to suggest that one of the greatest threats to conservation may be declining public support due to a progressively smaller population engaged in outdoor recreation and as a result less willing to support conservation activities. We analyze here the relationship between nature participation and future conservation support. Instead of treating all nature participation as equal, we ask if certain types of outdoor recreation are more tightly correlated with support for conservation than other types of recreation. Obviously the amount of support for conservation is also a function of broader economic forces [6]. As a result, the current economic crisis has caused enormous reductions in conservation effort [7]. However the time series we investigate were developed before the current economic downturn manifested its impacts.

## Methods

The nature exposure time series (Table 1) we chose are updated from those utilized in Pergams and Zaradic [4]. The conservation support variables we chose are contributions to four large conservation NGOs (The Nature Conservancy, World Wildlife Fund, Sierra Club, and Environmental Defense); updated from Pergams et al. [6]. Conservation contributions reflect the relative priorities of the donors, as well as the state of the US national economy and changes in both corporate and personal income and wealth [6]. As appropriate, values were weighted *per capita* and/or inflation-adjusted to 2001 dollars using the appropriate time series of GDP deflators from the US Federal Reserve Bank.

**Table 1:** Abbreviations, periods, definitions, and sources of nature variables used in this paper.

	Variable	Period	*N*	Definition	Data Source
**Public Lands Visitation**	BLMV	1982–2005^a^	19^b^	(Total recreational visits to all US BLM properties)/(Total US population)	US Bureau of Land Management & http://www.census.gov/
	NFV	1939–2002	61	(Total recreational visits to all US National Forests)/(Total US population)	US National Forest Service & http://www.census.gov/
	NPV	1939–2005	67	(Total recreational visits to all US NPS properties)/(Total US population)	http://www.census.gov/statab/www/ & http://www.census.gov/
	SPV	1950–2003	24	(Total recreational visits to all US State Parks)/(Total US population)	Statistical Abstracts of the USA http://www.census.gov/statab/www/ & http://www.census.gov/
**Game Licenses**	Ducks	1935–2006	72	(Total no. of duck stamps issued)/(Total US population)	Ducks Unlimited & http://www.census.gov/
	Fishing	1950–2005	53	(Total no. of fishing licenses issued)/(Total US population)	Statistical Abstracts of the USA http://www.census.gov/statab/www/ & http://www.census.gov/
	Hunting	1950–2005	52	(Total no. of hunting licenses issued)/(Total US population)	Statistical Abstracts of the USA http://www.census.gov/statab/www/ & http://www.census.gov/
**Camping**	Camping	1970–2003	15	(No. of people surveyed that went camping anywhere over the past year)/(Total no. of people surveyed)^c^	Statistical Abstracts of the USA http://www.census.gov/statab/www/ & http://www.census.gov/
	mmCampingNP/NF	1988–2005	18	(No. of people surveyed that went camping in National Parks or Forests over the past year)/(Total no. of people surveyed)^c^	Mediamark, Inc.
	mmCampingSP/SF	1988–2005	18	(No. of people surveyed that went camping in State parks or Forests over the past year)/(Total no. of people surveyed)^c^	Mediamark, Inc.
**Backpacking/Hiking**	ATHiking	1935–2005	71	(No. of hikers completing all 3500 km of the Appalachian Trail)/(Total US population)	http://www.appalachiantrail.org/hike/thru_hike/facts.html &http://www.census.gov/
	Hiking	1970–2003	16	(No. of people surveyed that went hiking anywhere over the past year)/(Total no. of people surveyed)^c^	Statistical Abstracts of the USA http://www.census.gov/statab/www/ & http://www.census.gov/
	Backpacking	1972–2003	15	(No. of people surveyed that went backpacking anywhere over the past year)/(Total no. of people surveyed)^c^	Statistical Abstracts of the USA http://www.census.gov/statab/www/ & http://www.census.gov/
	mmBackpackingHiking	1988–2005	18	(No. of people surveyed that went backpacking or hiking anywhere over the past year)/(Total no. of people surveyed)^c^	Mediamark, Inc.

a Data exists for 1975–1981, but we were advised by BLM (T. McDonald, *pers. comm.*) that while 1982 and later data were based on reported use at fee sites and recreation concessions, data prior to 1982 was not and was much less reliable.

b BLM data does not exist for the four years 1990 and 1993–1995: this is not our omission.

c Weighted by survey firm to best estimate demographics of US population.

Most of the variables were not normally distributed, so Spearman rank-order correlation analyses were performed on all variables. Though it was our hypothesis that components of nature exposure are associated with conservation effort in the future, we had no idea what time lags might be involved. Accordingly, we designed an iterative subroutine in R v. 2.6.1 Patched (R Foundation for Statistical Computing 2008) that performed correlations on all possible time lags in years (a total of over 300 paired comparisons). In addition to the raw data correlations, we performed correlations using the % year-to-year change for each of the datasets (difference model [4]), a total of 307 comparisons. Our time lags were limited to approximately 25 years due to the limits of our NGO investment data.

We applied a stringent double filter to identify significant correlations: the correlation for any time series must be both significant and of the same polarity in both raw data and % year-to-year change data for identical lag years. In addition we arbitrarily discarded any correlations with very small N<3. Given the number of correlation comparisons, and to adjust for multiple comparisons, we designed a technique to evaluate the likelihood of spurious results using a randomization approach. We generated time series of the same length as our data, but drawn from a random uniform distribution. The random raw dataset was drawn from the standard Mersenne-Twister algorithm, which generates positive real integers. The random % change data was generated from Mersenne-Twister set to a uniform distribution between -1 and 1. We applied the same double-filter approach for the given number of doubled comparisons (307) and length of datasets (up to 70 years) in our lagged time series, and found the number of spurious results detected by random chance alone to equal zero. Expanding the randomization to 1838 doubled comparisons produced only three significant results at the *p*<0.05 level. Thus the likelihood of spurious correlations that are both significant and of the same polarity in both the raw data and the % year-to-year change data for identical lag years is highly unlikely (*p* = 0.0016). Moreover, spurious correlations at levels more significant than *p*<0.05 are substantially unlikely.

## Results

**Figure 1:**
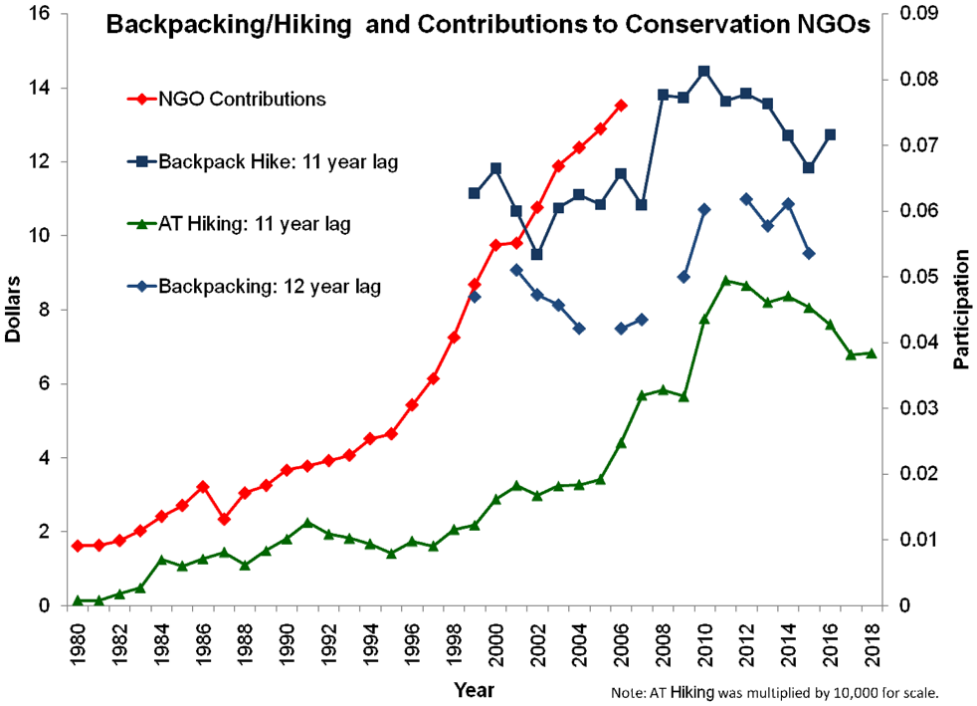
Comparing annual per capita contributions to conservation NGOs and participation in backpacking and hiking. Gaps in the backpacking time series are due to lack of data for those years. “NGO contributions” is aligned with actual year of contribution on the x-axis. The backpacking and hiking series are shown lagged by +11 or +12 years to match the significantly correlated year of NGO revenue. In other words, data for “NGO Contributions” for 2006 are depicted on the graph at x-axis year 2006 along with the correlated data for “Backpack Hike” from 1995, “AT hiking” from 1995, and “Backpacking” from 1994. Based on the correlation between these variables we would project a decline in NGO contributions until at least 2018.

Some nature exposure had a positive correlation with NGO contributions, and other types of nature exposure had negative correlations with NGO contributions (Table 2). Contributions are positively correlated with *per capita* backpacking and hiking 11–12 years earlier, but are negatively correlated with less elite outdoor activities, such as public lands visitation or fishing. Correlations with these latter forms of nature exposure are clumped into two time frames: a few years after the outdoor nature recreation occurs, and then approximately 16 years after the event. A chart comparing *per capita* annual conservation NGO contributions with *per capita* backpacking and hiking time series is given in Figure 1. The backpacking and hiking series are shown lagged in time to match the significantly correlated year of investment. Backpacking/hiking time series show longer-term increases, but with peaks 1998–2000 and declines since then.

**Table 2:** Results of Spearman correlations between various types of nature exposure (1^st^ column) and conservation NGO revenues.

Type of Nature Exposure	Category	Correlation With Conservation NGO Revenues (*ρs*)	Lag time (years)	Pos./Neg. Correlation	*P*	*N*
State Park Visitation	Visitation	−0.74545	4	Neg.	*	15
National Park Visitation	Visitation	−0.51892	4	Neg.	*	27
National Park/National Forest Camping	Visitation	−0.53431	7	Neg.	*	12
Backpacking/Hiking (Mediamark)	Hiking	0.80588	11	Pos.	***	8
Appalachian Trail Hiking	Hiking	0.61538	11	Pos.	*	27
Backpacking (Statistical Abstracts)	Hiking	0.90000	12	Pos.	*	6
Bureau of Land Management Visitation	Visitation	−0.67273	15	Neg.	*	9
National Park Visitation	Visitation	−0.85000	17	Neg.	**	27
Fishing	Visitation	−0.80952	17	Neg.	*	27

* indicates significant at 0.05 level, ** indicates 0.01 level, *** indicates 0.001. Correlations were performed for all possible lag periods in years. For example, results in the 1^st^ row (State Park Visitation) indicate that there was a significant, negative correlation between state park visitation and conservation NGO revenues 4 years later (Spearman's *rho*  = −0.74545, *P*<0.05, *N* = 15 comparisons. As state park visitation increased, NGO revenues decreased 4 years later. Conversely, as time spent backpacking/hiking (taken from the Mediamark series) increased, NGO revenues increased 11 years later. The table illustrates that the effect of public lands visitation (including fishing, shaded areas) had a negative effect on NGO revenues with two distinct time lags, 4–7 years later and 15–17 years later; while backpacking/hiking had a positive effect on NGO revenues 11–12 years later.

The declines in backpacking/hiking since 2000 could imply a significant problem for conservation support. Backpacking is defined in the survey data as 1+ overnight trips including wilderness camping, so backpackers require time and gear, both of which are relatively expensive. The two sets of survey data for backpacking, and the most extreme version of backpacking (complete thru-hiking of the 2000+ mile Appalachian Trail) are measures of similar, exclusive, high cost and time-intensive nature recreation. Backpacking tends to be more attractive to younger age groups. Most backpackers are in the 25–44 age range and overwhelmingly European-American [8], [9]. Using the most parsimonious interpretation of the time lags, these primarily European-American former backpackers become a significant source of NGO dollars 11–12 years after backpacking.

## Discussion

Our results show correlations between the type and timing of nature exposure and amount of later conservation investment. Our interpretation is that there are effectively two Americas when considering the pathway from nature exposure to conservation support: an elite backpacking/hiking group and a broader public lands visitation group. If this is true, then it has profound consequences for future generations and prospects for conservation support. Conservation organizations seem to be receiving donations from a very narrow group of relatively elite outdoor enthusiasts. The three variables which were highly positively correlated with conservation NGO contributions are all variations of backpacking (Table 2). Backpacking represents the least popular of the four classes of recreation variables studied [4]. The current per capita rate of backpacking is 0.054: in other words, the average American goes backpacking once every 18.5 years [4]. Each hiker or backpacker translates to $200–$300 annually in future NGO contributions. Given that most backpackers are 25–44 and adding the 11–12 year time lag, this would most likely be in middle age, presumably near their income prime. The demographics of this group are consistent with the description of the small fraction of the electorate that considers the environment a top priority: overwhelmingly European-American, mostly college educated, higher income and over 35 [2]. Further, based on the lagged impact of hiking/backpacking on investment, conservation NGOs have been benefiting from the tail end of a decade-old rise in the popularity of backpacking and hiking (Figure 1). The most recent data show a decline in hiking/backpacking popularity since 1998–2000 [4]. We project the negative effect of reduced hiking/backpacking frequency on NGO revenues to begin in approximately 2010–2011, and to continue through at least 2018 (Figure 1).

In contrast to backpacking, nature recreation at public lands appears much less likely to translate to conservation NGO investment. Moreover, there is apparently relatively little backpacking and hiking occurring during public lands visitation. For example, a recent survey of 849 Yellowstone National Park visitor groups asked what primary activities were their reasons for visiting the park [10, Fig. 43]. Seven activities accounted for 92% of reasons: Sightseeing/Taking a scenic drive (59%), Viewing wildlife/Bird watching (16%), Boardwalk/Geyser Basin (9%), camping in developed campgrounds (3%), Day hiking (3%), Viewing roadside/trailside exhibits (1%), and Overnight backpacking (backcountry) (1%). All other activities were <1% of reasons. With backpacking and hiking combined being only 4% of the reasons people gave for visiting Yellowstone, there is probably little ambiguity between public lands visitation data and data acquired specifically about backpackers and hikers.

In addition, there appears to be a large income gap of (very roughly) 30–70% between backpacker/hikers and public lands visitors. Based on a 30-year review of outdoor recreation literature by the USDA [11], average income (in 1996 inflation-adjusted dollars) of backpacker/hikers is $70,222. In contrast, the median household income for state and national park visitors (in 1996 inflation-adjusted dollars) is $42,100 to $44,438 [12]. The average income of sport fishermen of $43,410 is comparable to that of park visitors. Similar income gaps can be detected using market surveys of specialty magazines: Field and Stream for fishing/hunting (2008 annual circulation 16,500,000) has an average reader income of $54,838 [13a]; while Backpacking Magazine for hiking/backpacking (circ. 2,790,000) has an average reader income of $70,950 [13b].

Increased exposure to nature through public land visits (State Parks, National Parks, National Forests and Bureau of Land Management lands) is significantly negatively correlated with conservation NGO contributions (Table 2). Fishing is strongly associated with public lands; typically 80%–85% of all fishing takes place on public water bodies [14]. People who get their primary nature exposure from visiting public lands or fishing are much more diverse in age and ethnic composition [8], [9]. For example, in the African American community, fishing is more than seven times as popular (in *per capita* participation) than backpacking and more than twice as popular among the Hispanic community [9]. Similarly, visiting national parks is approximately three times more popular among Hispanics and four times more popular among the African American community as compared to backpacking [8]. The negative correlation between public land visitation and NGO contributions appears to hold both for short-term conservation investment (4–7 years after the nature experience) and for the longest-term measures we could obtain (approx. 16 years later). We hypothesize that people are more likely to invest in what they know from firsthand experience. Indeed it may be that high levels of public land recreation might create a sense that access to the outdoors is what is important rather than preserving less accessible landscapes through conservation NGO's. Whatever the reason, it is important to acknowledge that all nature participation is not equal in terms of generating support for conservation, and that to the extent that conservation needs to broaden its support base, there is a need to understand what type of nature recreation creates the strongest commitment to conservation.

Given the trends of increasing US population diversity, urbanization, and economic and cultural changes, we fear that the currently narrow base of conservation NGO supporters will become even narrower. To avoid becoming marginalized, the conservation movement will need to diversify its outreach strategy, engaging novel and diverse constituencies. Strategies for doing so may either require more of the “right types” of nature exposure, or entirely different approaches to ethnic or socioeconomic groups who are not likely to engage in hiking and backpacking. Ultimately, the fate of biodiversity and intact ecosystems may depend less on rates of habitat loss or invasive species, than on public perception of whether conservation should be supported at all.
